# Females know better: Sex‐biased habitat selection by the European wildcat

**DOI:** 10.1002/ece3.4442

**Published:** 2018-08-29

**Authors:** Teresa Oliveira, Fermín Urra, José María López‐Martín, Elena Ballesteros‐Duperón, José Miguel Barea‐Azcón, Marcos Moléon, José María Gil‐Sánchez, Paulo Celio Alves, Francisco Díaz‐Ruíz, Pablo Ferreras, Pedro Monterroso

**Affiliations:** ^1^ CIBIO/InBIO, Centro de Investigação em Biodiversidade e Recursos Genéticos Universidade do Porto Vairão Portugal; ^2^ Departamento de Biologia Faculdade de Ciências Universidade do Porto Porto Portugal; ^3^ Gestión Ambiental de Navarra Pamplona Navarra Spain; ^4^ Secció d'Activitats Cinegètiques i Pesca Continental Serveis Territorials de Barcelona Department of D'Agricultura, Ramaderia, Pesca i Alimentació Generalitat de Catalunya Barcelona Spain; ^5^ Wildlife Ecology and Health Group Facultat de Veterinària Universitat Autònoma de Barcelona (UAB) Bellaterra Barcelona Spain; ^6^ Agencia de Medio Ambiente y Agua Consejería de Medio Ambiente y Ordenación del Territorio Junta de Andalucía Granada Spain; ^7^ Department of Zoology University of Granada Granada Spain; ^8^ Wildlife Biology Program University of Montana Missoula Montana; ^9^ Biogeography, Diversity and Conservation Research Team Department of Animal Biology Faculty of Sciences University of Malaga Malaga Spain; ^10^ Instituto de Investigación en Recursos Cinegéticos (IREC, CSIC‐UCLM‐JCCM) Ciudad Real Spain

**Keywords:** European wildcat, resource selection, sex‐biased habitat selection, space use, spatial behavior

## Abstract

The interactions between animals and their environment vary across species, regions, but also with gender. Sex‐specific relations between individuals and the ecosystem may entail different behavioral choices and be expressed through different patterns of habitat use. Regardless, only rarely sex‐specific traits are addressed in ecological modeling approaches. The European wildcat (*Felis silvestris silvestris*) is a species of conservation concern in Europe, with a highly fragmented and declining distribution across most of its range. We assessed sex‐specific habitat selection patterns for the European wildcat, at the landscape and home range levels, across its Iberian biogeographic distribution using a multipopulation approach. We developed resource selection functions in a use‐availability framework using radio‐telemetry data from five wildcat populations. At the landscape level, we observed that, while both genders preferentially established home ranges in areas close to broadleaf forests and far from humanized areas, females selected mid‐range elevation areas with some topographic complexity, whereas males used lowland areas. At the home range level, both females and males selected areas dominated by scrublands or broadleaf forests, but habitat features were less important at this level. The strength of association to habitat features was higher for females at both spatial levels, suggesting a tendency to select habitats with higher quality that can grant them enhanced access to shelter and feeding resources. Based on our results, we hypothesize that sex‐biased behavioral patterns may contribute to the resilience of wildcats’ genetic integrity through influencing the directionality of hybridization with domestic cats. Our study provides information about European wildcats’ habitat use in an Iberian context, relevant for the implementation of conservation plans, and highlights the ecological relevance of considering sex‐related differences in environmental preferences.

## 1 INTRODUCTION

Human activities have widespread impacts across the environment and generally have disruptive effects in the ecosystems causing substantial threats to species diversity and conservation (Pimm et al., 2014). Therefore, a thorough understanding of how species respond to these changes is at the core of ecology and conservation biology. These studies can be addressed across temporal and/or spatial dimensions, and the latter is usually tackled through geographical (i.e., home ranges; Moorcroft, 2012) and carrying capacity approaches (i.e., resource selection; Manly, McDonald, Thomas, McDonald, & Erickson, 2004). Studying species’ spatial requirements contribute not only to the advancement of our understanding of their ecology but may also inform adequate management actions and allow the adoption of efficient conservation policies (Van Moorter, Rolandsen, Basille, & Gaillard, 2016).

Sexual differences in spatial behavior, particularly in the natal and breeding dispersal processes in mammals and birds, have been deeply investigated for decades, and the underlying causes for these differences have long been the subject of careful attention (see Dobson, 1982 and Greenwood, 1980; reviewed by Dobson, 2013). Less consideration, however, has been given to differences in habitat selection patterns between genders in established populations. Regardless, significant intersexual differences in habitat use patterns have been demonstrated in several mammal species. In northern Botswana, Stokke and Du Toit (2002) found that, during the dry season, family groups of African elephants (*Loxodonta africanus*) selected habitats closer to perennial drinking water than bull groups. In a species deeply affected by predation, the white‐tailed deer (*Odocoileus virginianus*), Kie and Bowyer (1999) found that females with young made greater use of habitats with dense cover than did males, where preferred herbaceous forage was less abundant. Carnivore species may also show distinct social behaviors and spacing patterns between genders (Crook, Ellis, & Goss‐Custard, 1976). Studies performed in several species of the Felidae family also suggest that females tend to prefer habitats that grant them higher protective cover and access to feeding resources (see Chamberlain, Leopold, & Conner, 2003; Conde et al., 2010 and Ramesh, Kalle, & Downs, 2015). Taken together, these studies are indicative of a general strategy across mammalian species by which females maximize their reproductive output and survival of the young. However, this topic remains largely underexplored in the scientific literature, potentially undermining a broader perception of the mechanisms driving social structure and demographic patterns of many species, with potential consequences for their management and conservation.

**Figure 1:**
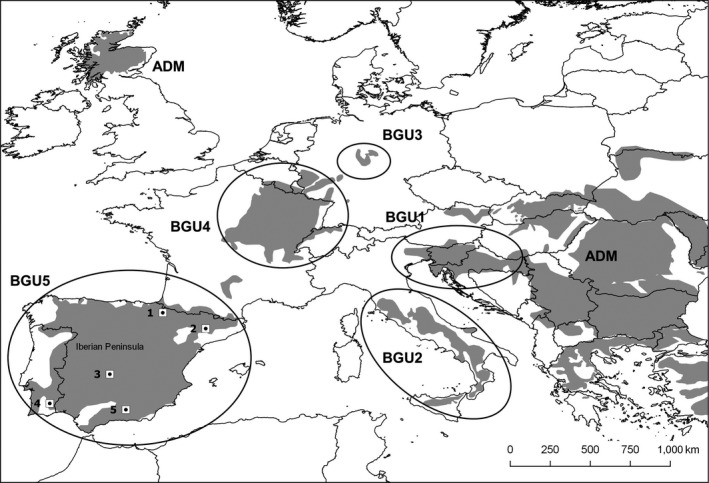
Location of our study areas within the distribution range of the European wildcat (*Felis silvestris silvestris*), adapted from IUCN Red List of Threatened Species, version 2013.2 (http://www.iucnredlist.org), and the five European wildcat biogeographic groups (adapted from Mattucci et al., 2016): BGU1—eastern and Dinaric Alps, BGU2—Italian peninsula and Sicily, BGU3—central Germany, BGU4—France, Belgium, Luxembourg, Switzerland, and southwestern Germany, BGU5—Iberian Peninsula, ADM‐ Samples from eastern Europe (Poland, Bulgaria and Hungary) and Scotland corresponded to highly admixed or introgressed individuals, and therefore where not assigned to any specific BGU. 1—Izagaondoa Valley (IZV); 2—the Lleida region (LD); 3—Cabañeros National Park (CNP); 4—Guadiana Valley Natural Park (GVNP), 5—Sierra de Arana (SA)

Despite being classified as “Least Concern” at global scale, the European wildcat (*Felis silvestris silvestris*, Schreber, 1777) is a protected flagship species of special conservation concern in Europe, where it is strictly protected through the Bern Convention and Habitats Directive (Yamaguchi, Kitchener, Driscoll, & Nussberger, 2015). A recent study reported five main genetic clusters in Europe, corresponding to distinct biogeographic units (BGUs, Figure 1), and all except the Iberian BGU exhibit eroded within‐cluster genetic diversity, most likely due to recent bottlenecks (Mattucci, Oliveira, Lyons, Alves, & Randi, 2016). These results support not only the conservation importance of the Iberian wildcat metapopulation but also suggest it was a stronghold in the recent history of the wildcat in Europe. However, declines across several wildcat populations in Iberia have been reported (Lozano, Virgós, Cabezas‐Díaz, & Mangas, 2007; Sarmento, Cruz, Eira, & Fonseca, 2009; Sobrino, Acevedo, Escudero, Marco, & Gortázar, 2008), which prompted its conservation status to “Vulnerable” in Portugal (Cabral et al., 2005), and “Near Threatened” in Spain (López‐Martín et al. 2007). Moreover, the recent emergence of a new variant of the rabbit hemorrhagic disease virus (*Lagovirus europaeus*/GI.2) is reducing the availability the European wildcats’ main prey in the Mediterranean ecosystems (Monterroso et al., 2016). Another threat to the conservation of European wildcats is hybridization with con‐specific domestic cats (*F. silvestris catus*), which is documented throughout Europe (Mattucci et al., 2016; Yamaguchi et al., 2015). These recent population declines, prey scarcity and widespread hybridization, coupled with habitat degradation, depict a concerning conservation scenario for the Iberian wildcat BGU. Therefore, a robust understanding of European wildcats’ ecological requirements and threats is paramount to defining sound conservation plans (Lozano & Malo, 2012). The Iberian wildcat BGU encompasses several bioclimatic regions, suggesting a high degree of ecological flexibility (Bolnick, Svanback, Araujo, & Persson, 2007). Therefore, local‐scale studies performed so far (e.g., Lozano, Virgos, Malo, Huertas, & Casanovas, 2003; Monterroso, Brito, Ferreras, & Alves, 2009; Sarmento, Cruz, Tarroso, & Fonseca, 2006) may confound our broad understanding of the species’ ecological patterns across the wider Iberian BGU.

Moreover, most habitat selection studies still ignore sex‐related differences, potentially underestimating important environmental factors for one sex and overemphasizing them for the other (Conde et al., 2010). Such differences could entail important consequences for the European wildcat as they may reveal sex‐specific behavioral responses to human‐induced environmental change, deepening our understanding of this species’ adaptive and nonadaptive responses, and potentially unraveling key aspects related to animal's ability to cope with novel environments (Sih, 2013; Sih, Ferrari, & Harris, 2011). For example, if females have a narrower spatial niche breadth than males, then their capacity to adapt to habitat changes (e.g., changes in land use) might be limited.

Another fundamental aspect of wildcats’ behavior with high relevance for the species’ conservation is the contact with domestic cats, potentially leading to hybridization and disease transmission (such as Feline leukemia virus, FeLV, and feline immunodeficiency virus, FIV). Different space use patterns could influence hybridization directionality (defined as the relative skewness in the frequency of interbreeding events between two groups under reciprocal hybridization; Bettles, Docker, Dufour, & Heath, 2005), and consequently the incorporation of hybrids into the wild population. This situation was reported for canid species—grey wolf x dog (Godinho et al., 2011; Leonard, Echegaray, Randi, & Vilà, 2013), or red wolf x coyote (Bohling & Waits, 2015)—, where matings between female wolves and male dogs, and between female red wolves and male coyotes, were overwhelmingly more abundant than the opposite. These examples illustrate how the behavior and interbreeding processes can have important consequences for the conservation of fragile populations. Therefore, considering sex‐specific behavioral patterns across a broad geographic scale is of utmost importance for accurately defining conservation strategies, particularly for the European wildcat in the Southwestern part of its range, where it is currently of high conservation concern (Cabral et al., 2005; López‐Martín et al. 2007).

With this study we evaluate sex‐specific patterns of space use by European wildcats. Specifically, we estimate home range sizes and develop sex‐specific resource selection functions (RSFs) for the European wildcat, to test a hypothesis of sex‐biased habitat selection. We hypothesize that females should select higher quality habitats to secure adequate breeding grounds and resource availability within smaller areas, whereas males should be more habitat‐flexible so as to gain access to multiple females, occupying larger territories. We develop RSFs at the landscape and home range levels across several populations within the range of the Iberian wildcat BGU. With this broad scale population‐structured approach, we provide new insights into the spatial ecology of the European wildcat in southwestern Europe, which can inform conservation strategies aimed at reversing the declining trends of this small felid.

## 2 MATERIAL AND METHODS

### 2.1 Study areas and European wildcat data

European wildcats were captured and radio‐tracked in five study areas across the Iberian Peninsula (Figure 1): the Guadiana Valley Natural Park (GVNP; SE Portugal, ~37°41.4′N, 7°45.6′W), the Izagaondoa Valley (IZV; NE Spain, ~42°46.8′N, 1°25.2′W), the Lleida region (LD; NE Spain, ~41°31.2′N, 1°48.0′E), the Cabañeros National Park (CNP, C Spain, ~39°19.8′N, 4°23.4′W), and Sierra Arana (SA; SE Spain, ~37°20.4′N, 3°29.4′W). The GVNP, LD, CNP, and SA are located in the Mediterranean biogeographic region, whereas IZV is in the transition between the Mediterranean and Atlantic European biogeographic regions (Rivas‐Martínez, Penas, & Díaz, 2004) (Figure 1). The natural vegetation at the GVNP, CNP, and LD is dominated by scrublands of *Cistus* spp., and the tree layer is dominated by cork oaks *Quercus suber*, holm oaks *Quercus rotundifolia,* and pine plantations of *Pinus* spp. Agroforestry systems can also be found with native grassland understory (CNP) or cereal cropland understory (GVNP). Agricultural areas of cereal crops have a relevant expression at LD's landscape. The landscape at SA is dominated by Aleppo pine (*P. halepensis*) forests with patches of cork and holm oaks. At higher altitudes, forests with scrubland understory are interspersed with dense scrubland patches, mainly composed by juniper (*Juniperus oxicedrus*), rosemary (*Rosmarinus officinalis*), furze (*Ulex parviflora*), and grey‐leaved cistus (*C*. *albidus*). Broadleaf forests characterize the natural vegetation at higher altitudes at IZV, with a strong presence of the common beech (*Fagus sylvatica*). At lower altitudes, the common beech is replaced by forests of pubescent oak (*Q. pubescens*), with holm oak predominating in xeric areas. Scrubland areas are dominated by boxwood (*Buxus sempervirens*) and *Genista scorpius* bushes, sometimes interspersed with patches of herbaceous vegetation and former agricultural areas. An ecological overview of the study areas is provided in Supporting Information Appendix S2.

Domestic cats occur in all study areas (except in CNP, where their occurrence is reduced), mainly associated to human settlements and its close vicinities. However, all individuals were analyzed using genetic markers and revealed no trace of admixture with domestic cats (see Supporting Information Appendix S1). Information regarding capture and monitoring procedures of European wildcats for each study area is provided in Supporting Information Appendix S1. All applicable institutional and/or national guidelines for the care and use of animals were followed.

### 2.2 Home range estimation

We estimated individual wildcats’ home ranges using Kernel Density Estimators (KDEs) at the 90% isopleth using a fixed reference scaled bandwidth, which was estimated manually by reducing the reference bandwidth by steps of 0.1 until the home range became fragmented or presented lacuna (Kie, 2013). The minimum number of fixes required for reliable home range estimation was determined with a bootstrap analysis, by estimating the minimum convex polygon area (MCP), increasing the sample size logarithmically until the MCP achieved an asymptote (Kenward, 2000). All home range analyses were performed using R software v3.2.5 (R Core Team 2017) with the *rhr* v1.2.906 package (Signer & Balkenhol, 2015). A default output grid of 100 × 100 was used to perform home range analyses. Bootstrap analyses were performed using *move* v2.1.0 R‐package (Kranstauber & Smolla, 2016), with 100 repetitions per step. Differences between male and female home range sizes were investigated with type II ANOVA tests, using *stats* v3.4.3 package (R Core Team 2017). Data on home range size was square‐root transformed to achieve normality prior to analysis. The normality of the response variable and ANOVA residuals was checked using the Shapiro–Wilk test.

### 2.3 Predictor covariates

We considered three types of covariates potentially important for the European wildcat: landcover, topography, and disturbance‐related (Table 1). Although we acknowledge that prey availability is a potentially important covariate group, there was no such data available for analysis across all study sites. Therefore, we were unable to consider it in our models. Landcover data were obtained from SIOSE for the Spanish study sites: SIOSE 2005—for IZV, LD, and SA—and 2011—for CNP (http://centrodedescargas.cnig.es/CentroDescargas/index.jsp#); and COS2007 (available at http://www.igeo.pt/DadosAbertos/Listagem.aspx#;) for GVNP (Portugal) so that landcover layers matched the location and sampling period as much as possible. The original datasets were reclassified into eight ecologically relevant landcover classes for the European wildcat, based on the published literature (e.g., Lozano et al., 2003; Monterroso et al., 2009; Sarmento et al., 2006): agricultural, agroforestry systems, broadleaf forests, coniferous forests, mixed forests, scrublands and transitional woodland scrub, natural herbaceous vegetation, and open areas. Only vegetation classes with availability above 5% within home range limits and study areas were considered for analysis (Palomares et al., 2000). Specific landscape features, such as human structures (human settlements and roads) and water sources, might have disproportionally large effects in shaping the spacing patterns of the European wildcat (e.g., Klar et al., 2008; Monterroso et al., 2009). Therefore, we included the distance from each telemetry fix to the nearest feature of each of these covariates as potential explanatory covariates (Table 1), despite their low representativity in the study areas. Due to a poor representation of these features in the main landcover datasets, we extracted vector layers of human settlements, roads, and permanent water bodies from *OpenStreetMap* (OpenStreetMap contributors, 2018). The remaining landcover covariates consisted on the Euclidean distance from each telemetry fix to each landcover class, as well as the area occupied by each class within a 150‐m radius circular buffer centered at each telemetry fix (or randomly generated point, see below) (Table 1). We used 150‐m buffer size as previous studies have shown that wildcats respond to landscape covariates at this spatial scale (Monterroso et al., 2009). We considered slope and elevation at each fix (or randomly generated point) as potentially relevant topographic covariates (Table 1). Slope information was derived from ASTER‐DGEM digital elevation models (DEMs), with a 30 × 30 m resolution (https://gdex.cr.usgs.gov/gdex/). All spatial analyses were performed using QGIS v2.14 vector tools (Quantum GIS Development Team 2016). Distances were obtained using GRASS GIS v7.0.3 vector tools (GRASS Development Team 2016). Grids for slope were created over the DGEM layer with QGIS v2.14. DEM tools.

**Table 1:** Covariates considered in the habitat selection models for European wildcats in the Iberian Peninsula

Type	Class	Description	Measure	Code	Females			Males		
					Available (Landscape level)	Available^a^ (Home range level)	Used (Home range level)	Available (Landscape level)	Available^a^ (Home range level)	Used (Home range level)
Landcover	Broadleaf forests	Patches with ≥30% of broadleaf forest cover, and undercover not used for agriculture	Area (ha)	BFr	1.42 ± 0.02	2.09 ± 0.06	0.90 ± 0.04	1.10 ± 0.01	1.07 ± 0.05	1.27 ± 0.05
			Distance to nearest edge (m)	dBFr	331.61 ± 4.45	308.50 ± 4.05	167.54 ± 11.09	446.05 ± 3.47	300.46 ± 16.68	301.37 ± 17.20
	Scrublands	Patches with ≥25% scrublands, and <30% forest cover	Area (ha)	Scr	1.69 ± 0.02	1.82 ± 0.05	2.13 ± 0.06	1.56 ± 0.01	1.56 ± 0.06	1.56 ± 0.06
			Distance to nearest edge (m)	dScr	154.27 ± 1.76	159.42 ± 1.87	118.16 ± 4.59	182.39 ± 1.48	141.92 ± 5.33	135.58 ± 4.82
	Agricultural areas	Patches with >50% agricultural activities	Area (ha)	Agr	1.82 ± 0.02	0.88 ± 0.04	0.90 ± 0.04	2.64 ± 0.01	2.68 ± 0.07	2.51 ± 0.07
			Distance to nearest edge (m)	dAgr	356.78 ± 5.67	418.66 ± 7.11	330.49 ± 9.27	330.46 ± 4.85	201.05 ± 11.40	177.80 ± 10.26
	Permanent water bodies	Water bodies available throughout the year (main rivers and streams)	Distance to the nearest edge (m)	dW	1,958.11 ± 16.85	3,025.40 ± 9.31	2,894.06 ± 28.45	1,632.57 ± 16.87	1,272.87 ± 12.50	1,160.52 ± 37.65
Disturbance	Human Settlements	Humanized areas (cities, villages	Distance to the nearest edge (m)	dH	1,318.62 ± 10.88	2,076.31 ± 19.69	1,896.97 ± 59.86	1,166.71 ± 10.67	1,711.82 ± 15.12	1,596.83 ± 46.35
	Roads	Major paved roads (primary, secondary, tertiary)	Distance to the nearest edge (m)	dR	1,315.59 ± 12.36	3,370.28 ± 17.70	3,240.78 ± 54.26	1,252.73 ± 12.19	1,575.82 ± 14.24	1,497.63 ± 42.45
Topographic	Slope	Steepness	Degrees	Slp	13.88 ± 0.07	13.79 ± 0.07	16.24 ± 0.22	11.42 ± 0.05	11.69 ± 0.24	11.86 ± 0.24
	Elevation	Height above sea level	Meters above sea level (m.a.s.l.)	Elev	597.73 ± 1.59	592.02 ± 1.51	598.02 ± 4.48	578.27 ± 1.28	530.81 ± 5.40	572.43 ± 5.48

The “available” sample for home range scale selection is the same as the “used” sample for landscape scale selection (see methods section).

### 2.4 Habitat selection analyses

We investigated habitat selection at two levels—landscape and home range—following the scalar hierarchy proposed by Johnson (1980), and examined selection at both levels using resource selection functions (RSFs; Manly et al., 2004). At the landscape level (Johnson's 2nd order selection), we compared two randomly generated sets of points: one generated within each animal's home range (“used” sample) and the other generated within the available landscape (“availability” sample). We considered the available landscape as the minimum convex polygon taken from outermost fixes for each studied population, to which we added a buffer equal to the mean home range radius of our radio‐tracked animals, *r =* 2.11 km (Dillon & Kelly, 2008). At the home range level (Johnson's 3rd order selection), we compared radio‐tracking locations from each animal within its respective home range (“used” sample) with the set of random points within its home range generated in the previous step (“availability” sample), therefore, applying a two‐level hierarchical approach. The number of random points generated to represent availability was ten times the number of fixes used for each individual and population, for both selection levels of analysis (Koper & Manseau, 2012; Northrup, Hooten, Anderson, & Wittemyer, 2013).

We developed RSFs for males and females independently, at both levels, to account for potentially different habitat selection patterns. We developed generalized linear mixed‐effects models (GLMMs) with a dummy response variable representing used and available locations (1 and 0, respectively), fitted with a logit link function. Individual wildcats were considered as random effect to accommodate for inter‐individual and uneven sample size variation (Gillies et al., 2006). Although we considered that study areas could be a potential source of variation, preliminary analyses showed that including individuals wildcats as the sole random variable provided the most parsimonious models. We developed RSFs following a sequential stages approach. First, we assessed the univariate association between the response variable and each covariate in their linear and quadratic forms. The complete set of univariate models for each covariate class was ranked according to their Akaike's information criteria corrected for small sample size (AICc) (Burnham & Anderson, 2002), and we retained the covariates in the model with the lowest AICc value. We then conducted a Spearman rank correlation test on all retained covariate pairs. Whenever a pair of covariates was correlated (ρ > 0.5), we kept that which had a stronger univariate relationship with our response variable (Hosmer & Lemeshow, 2000). On a second stage, we developed a full‐effects model which included all covariates retained in the previous step. Then, we built a set of models with all possible combinations between covariates, which were then ranked following AICc criteria (Burnham & Anderson, 2002). We considered models with ΔAICc values ≤2 units of the lowest AICc to have substantial support for being best models (Burnham & Anderson, 2002). The coefficients of each variable included in the top models’ set were assessed following a model averaging procedure (Burnham & Anderson, 2002). All continuous variables were standardized to z‐scores prior to modeling. Models that failed to converge were excluded from the analyses.

Model fit was assessed using k‐fold cross‐validation whereby 80% of the data was used in the modeling procedures, which were then used to predict the probability of use in the remaining 20% (Boyce, Vernier, Nielsen, & Schmiegelow, 2002). This procedure was repeated five times until all data had been used. Spearman rank correlations were used to evaluate the relationships between the frequency of cross‐validated used locations and 10 probability bins of equal size, representing the range of predicted values. A model with good predictive performance should show a strong correlation (ρ  >  0.80) (Boyce et al., 2002). All analyses were conducted using R version 3.2.5 (R Core Team 2017). All models and respective k‐fold cross‐validation were developed using lme4 v1.1‐12 R‐package (Bates, Maechler, Bolker, & Walker, 2015). AICcmodavg v2.0‐4 (Mazerolle, 2017) was used to perform model averaging, and MuMIn v1.15.6 (Barton, 2018) was used to create and rank all model combinations. Results are presented as mean ± *SE*, except when explicitly stated otherwise.

## 3 RESULTS

### 3.1 Home range size

We captured 31 European wildcats (16 males and 15 females), representing 6.20 ± 0.70 individuals per study area (Supporting Information Appendices S1 and S3). A total of 2,976 fixes were obtained, with an average of 96.00 ± 28.00 fixes/individual. We obtained reliable home range estimates for 18 wildcats (11 males and seven females), distributed across all study areas (Supporting Information Appendix S3). Therefore, only these individuals were considered for all posterior analyses. Home range size ranged from 1.22 to 59.78 km^2^ (90% Kernel isopleth) and presented a median of 13.68 km^2^. Males tended to have larger home ranges than females (median HR_males_ = 14.68 km^2^ [range: 1.22–43.01] vs. HR_females_ = 4.59 km^2^ [3.14–59.78]), although without statistically significant differences (*F*[1, 8] = 1.35, *p* = 0.26; W = 0.93, *p* = 0.19). However, home range size was statistically different among the five study areas (*F*[4, 8] = 4.20, *p* = 0.02; W = 0.95, *p* = 0.37).

### 3.2 Habitat selection at landscape and home range levels

**Figure 2:**
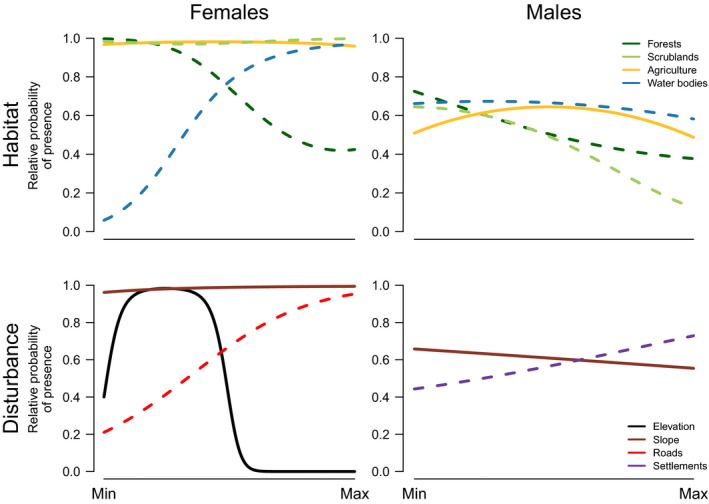
Predicted relative probability of male and female wildcat presence at the landscape (2nd order) level according to the top‐ranked resource selection functions (RSFs). Full lines and dashed lines correspond to the proportion of area occupied or distance to that habitat type, respectively. Covariate ranges: elevation [0–2,000 m.a.s.l.]; slope [0–60 degrees]; habitat type area [0–7 ha]; distance to habitat type [0–2,000 m]

We considered 2,671 locations (1,181 for males and 1,490 for females) obtained within home range limits to evaluate habitat selection. The RSF that best explained landscape‐level habitat selection by females included both linear and quadratic terms for agricultural and scrubland areas, for distance to broadleaf forests, and for slope and elevation (Table 2). The following model, within a ΔAICc ≤ 2, included also both the linear and quadratic terms for distance to roads and to permanent water sources (Table 2). At the same level, males obtained a single top‐ranked model with ΔAICc ≤ 2, which included both the linear and quadratic terms for agriculture, for distance to scrublands, broadleaf forest, human settlements, and permanent water bodies. Among topographic covariates, only the linear term for slope was included. Our RSFs suggest that females established home ranges away from paved roads and permanent water bodies, but close to broadleaf forests (<500 m), particularly at mid‐range elevation areas (300–800 m.a.s.l.) (Supporting Information Appendix S4, Table D3; Figure 2). Males established home ranges far from human settlements but closer to permanent water sources, broadleaf forests and scrublands, and with intermediate levels of agriculture (Supporting Information Appendix S4, Table D3; Figure 2). Interestingly, the strength of association/avoidance (i.e., coefficient estimates) with specific habitat features were higher for females than for males (Supporting Information Appendix S4, Table D3). The cross‐validation procedure revealed a poor and a good model fit for females and males final RSFs, respectively (ρ_females_ = 0.41; ρ_males_ =0.96; Table 2).

**Table 2:** Top‐ranked (ΔAICc < 2) RSFs for landscape and home range habitat selection (2nd and 3rd orders, respectively) by female and male European wildcats

Selection level	Gender	Model	*k*	ΔAICc	Weight	ρ
Landscape	Females	Agr +Agr^2^+ dBFr + dBFr^2^+ Scr +Scr^2^ + Slp + Slp^2^ + Elv + Elv^2^	16	0.00	0.56	0.41
		Agr + Agr^2^ + dBFr + dBFr^2^ + Scr + Scr^2^ + dR +dR^2^ + dW + dW^2^ + Slp + Elv + Elv^2^	15	0.54	0.43	0.41
	Males	Agr +Agr^2^+ dBFr + dBFr^2^+ dScr +dScr^2^ + dW + dW^2^ + dH + dH^2^ +Slp	13	0.00	0.99	0.96
Home range	Females	BFr + Scr + dW + dH	6	0.00	0.25	0.95
		BFr + Scr + dW + dH + dH^2^	7	0.85	0.17	0.99
		BFr + Scr + dW + dH + Slp	7	1.69	0.11	0.96
		BFr + Scr + Scr^2^ + dW + dH	7	1.89	0.01	0.94
	Males	Agr + dBFr + dScr + dScr^2^ + Slp + Slp^2^ + dW + dW^2^	10	0.00	0.09	0.75
		Agr + dBFr + dBFr^2^ + dScr + dScr^2^ + Slp + Slp^2^ + dW + dW^2^	11	0.04	0.08	0.71
		Agr + dBFr + dBFr^2^ + dScr + dScr^2^ + Slp + dW + dW^2^	10	0.05	0.08	0.80
		Agr + dBFr + dScr + dScr^2^ + Slp + dW + dW^2^	9	0.15	0.08	0.60
		dBFr + dBFr^2^ + dScr + dScr^2^ + Slp + Slp^2^ + dW + dW^2^	10	1.06	0.05	0.68
		dBFr + dScr + dScr^2^ + Slp + Slp^2^ + dW + dW^2^	9	1.65	0.04	0.77
		dBFr + dBFr^2^ + dScr + dScr^2^ + Slp + dW + dW^2^	9	1.78	0.04	0.71
		dBFr + dBFr^2^ + dScr + Slp + Slp^2^ + dW + dW^2^	9	1.86	0.03	0.82
		Agr + dBFr + dBFr^2^ + dScr + Slp + Slp^2^ + dW + dW^2^	10	1.90	0.03	0.77
		Agr + dBFr + dBFr^2^ + dScr + Slp + dW + dW^2^	9	1.93	0.03	0.76

Note

*k*: Number of model parameters; AICc: Aikake information criterion corrected for small sample sizes; ρ: Spearman rank correlation (see methods section); Agr: Area occupied by agricultural lands; dAgr: distance to agricultural lands; dBFr: distance to broadleaf forests; BFr: area occupied by broadleaf forests; Scr: area occupied by scrublands; dScr: distance to scrublands; Slp: Slope; Elv: elevation; dR: distance to roads; dW: distance to permanent water sources; dH: distance to human settlements.

**Figure 3:**
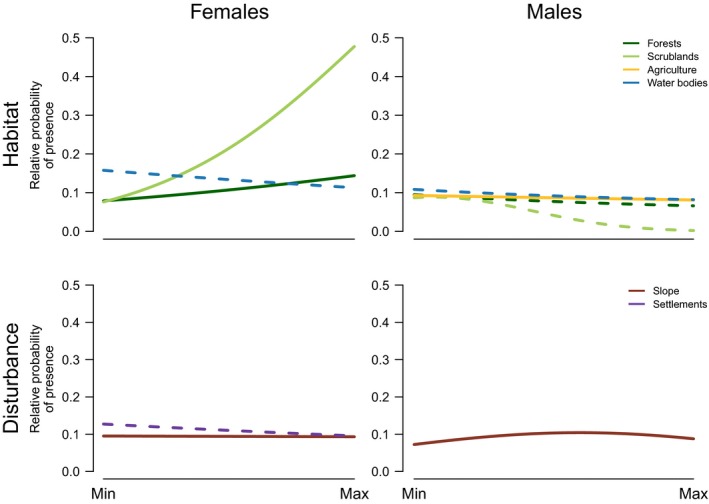
Predicted relative probability of male and female wildcat presence at home range (3rd order) level according to the top‐ranked resource selection functions (RSFs). Full lines and dashed lines correspond to the proportion of area occupied or distance to that habitat type, respectively. Covariate ranges: elevation [0–2,000 m.a.s.l.]; slope [0–60 degrees]; habitat type area [0–7 ha]; distance to habitat type [0–2,000 m]

The best RSF model explaining habitat selection at the home range level by female wildcats included the linear term for scrublands, broadleaf forests, and distance to human settlements and to permanent water sources. The next three models, with a ΔAICc ≤ 2, also included the quadratic term for human settlements, scrublands, and slope (Table 2). A set of 10 top‐ranked models emerged for males at the home range level, including combinations of the linear and quadratic terms of agricultural areas, distance to broadleaf forests, to scrublands and to permanent water bodies, and slope (Table 2). Females favored the use of areas with higher vegetation cover (scrublands and broadleaf forests) and closer to human settlements within their home ranges, whereas male wildcats selected steeper areas closer to water bodies and to broadleaf forests, but avoided agricultural fields (Figure 3). Although the strength of association with specific habitat features within home ranges was higher for females than for males, their importance appears to be lower at the home range than at the landscape level (Supporting Information Appendix S4, Table D3). The cross‐validation procedure revealed good model fit for both sexes (ρ_females_ = [0.94–0.99]; ρ_males_ = [0.60–0.82]; Table 2).

## 4 DISCUSSION

### 4.1 Home range size

We observed a high variability in home range size for European wildcats in the southwestern range of its distribution, both at the intraspecific and intragender levels. While the flexibility in male home range size reflects a pattern observed throughout its distribution range, we detected much higher variability than ever reported for females. Male home ranges are known to vary between 3.03 km^2^ (Anile et al., 2017) and 53.30 km^2^ (Liberek, 1999). Conversely, females’ have been reported to range between <1.5 km^2^ (Germain, Benhamou, & Poulle, 2008) and 6.23 km^2^ (Jerosch, Götz, & Roth, 2017). We found female wildcats’ home range size in the Iberian BGU to vary between 3.14 and 59.78* *km^2^ (mean ± *SE*: 13.56 ± 7.25 km^2^), one order of magnitude higher than previous reported in its entire European range. The largest female home range was recorded at CNP and is equivalent to the largest estimate among males (also found at this study site). The large home ranges found at this study site could be the result of a combination of limited prey availability (Ferreras, Díaz‐Ruiz, Alves, & Monterroso, 2016; Monterroso, Alves, & Ferreras, 2014) and suboptimal habitat quality for wildcats. Home range size of carnivores is related to metabolic needs, diet, habitat characteristics, and distribution of feeding resources, as well as interactions at both inter‐ and intraspecific levels (Gittleman & Harvey, 1982; Gompper & Gittleman, 1991). While having sampled European wildcats from five areas with intrinsically distinct characteristics provides a broader perception of the species requirements and ecological flexibility, it predictably entails the high variability that characterizes this species. Therefore, it is likely that the heterogeneity in habitat conditions, demographic parameters, and prey availability among study areas would be reflected in the observed variability in home range size, even among individuals of the same gender (Mattisson et al., 2013; Nilsen, Herfindal, & Linnell, 2016). This high variability observed in home range sizes led to a statistically nonsignificant difference between genders, although median values of home range areas for males were larger than for females, which was expected as it should reflect different selective pressures for reproductive success (Gehrt & Fritzell, 1998). This tendency has been reported in other wildcat BGUs (Anile et al., 2017; Liberek, 1999; Stahl, Artois, & Aubert, 1988), as well as in other solitary felids (Ferreras, Beltrán, Aldama, & Delibes, 1997; Herfindal, Linnell, Odden, Nilsen, & Andersen, 2005; Tucker, Clark, & Gosselink, 2008).

### 4.2 Sex‐biased habitat selection

Male and female European wildcats exhibited distinct habitat selection patterns, with several habitat components presenting a different importance for the establishment of both genders’ home ranges, thereby supporting our initial hypotheses. Our results support that females settle their home ranges in areas with protective cover and low disturbance, but that grant them access to predictably high prey availability habitats (i.e., scrubland‐agriculture mosaics). A reduced level of contact with anthropogenic features and permanent water sources (associated with lower elevations) revealed to be important, as disclosed by their high coefficient values. At the same biogeographic region, Sarmento et al. (2006) found that female wildcats selected autochthonous broadleaf forests and avoided dense scrubland habitats in a mountainous area with low rabbit availability. The authors argued that broadleaf forests provide shelter and the highest availability of rodents, the most consumed food resources locally (Sarmento, 1996). The apparent lower importance of these habitat variables for male wildcats may suggest that they are less influenced by habitat quality, hence, potentially more tolerant to habitat fragmentation and human contact.

Within home ranges, the strength of association to the considered covariates was lower than observed at the landscape level, which suggests a pattern of use closer to the covariates’ availability. Nevertheless, females retained a stronger association to habitat features than males. Females also selected areas that provided vegetation cover, although there was a tendency to use areas closer to human settlements, where they can gain access to agricultural fields (as interpreted by the high correlation between these two covariates: ρ = 0.60). This behavior could potentially be related to high prey availability in these areas. The lower strength of association at the home range level could be indicative of either habitat selection occurring primarily at the higher level, that is, landscape, or the existence of unaccounted covariates with higher importance for wildcats at this level. Prey availability has been widely reported to influence the distribution of this species (Klar et al. 2008, Monterroso et al., 2009; Silva, Kilshaw, Johnson, Macdonald, & Rosalino, 2013). The European rabbit is the main prey of European wildcat's in the southwestern distribution range, where rodents act as alternative prey (Lozano, Moleon, & Virgos, 2006). European rabbits benefit from a mosaic habitat structure that maximizes scrubland‐pastureland ecotone (Fernández, 2005; Lombardi, Fernández, Moreno, & Villafuerte, 2003); therefore, these areas provide the most profitable hunting grounds for European wildcats in Mediterranean areas. However, as rabbit availability decreases in more temperate or alpine climates, rodents take place as European wildcats’ main prey (Lozano et al., 2006). Rodents are also usually associated with mosaic habitats, namely at forest edges, although they are also present in agricultural areas, especially near streams (Osbourne, Anderson, & Spurgeon, 2005; Sullivan & Sullivan, 2006). Although we could not consider prey availability in our models, the habitat structure preferentially selected by European wildcats matches where prey abundance is predictably highest.

Sex‐biased habitat selection where females display stronger selection for better quality habitats is consistent with those observed in the other wild felids. Conde et al. (2010) observed that while both male and female jaguars (*Panthera onca*) showed selection for tall forests, females strongly avoided the disturbed habitats whereas males used them in proportion to their availability. Also, Broomhall, Mills, and du Toit (2003) found that female cheetahs (*Acinonyx jubatus*) used dense woodlands, which provided the greatest prey availability and protective cover against dominant competitors, more frequently than males. Chamberlain et al. (2003) found that male and female bobcats (*Lynx rufus*) selected and used habitats differently. Female bobcats consistently selected <8 year‐old pine stands, where prey is more abundant, and other habitats were even least selected during breeding and kitten‐rearing. This “prudent female” behavior is consistent with the sex‐biased selection pattern we observed in European wildcats. However, our habitat selection models for females at the landscape level performed poorly, with correlation values below 0.50, which may indicate some level of variability in habitat selection within this level. The inclusion of other covariates in our model, such as prey availability, could also provide a clearer picture about females’ home range selection process across the landscape.

### 4.3 Implications for the contact with domestic cats: hybridization and disease transmission

Paired with the reduction of suitable habitat, hybridization with domestic cats is one of the main conservation threats to European wildcats (Yamaguchi et al., 2015). Some studies (see Germain et al., 2008; Gil‐Sanchez, Jaramillo, & Barea‐Azcon, 2015; Sarmento et al., 2009) suggest that behavioral differences between wild and domestic cats could mediate a barrier between the two counterparts, which could be weakened by a depression of the wildcat population, with concomitant increased contact between these two subspecies.

Our results support that female wildcats have stricter habitat requirements than males, which appear to exhibit higher tolerance to habitat fragmentation and human presence. This setting suggests that females might be determinant in maintaining the cohesion of natural wildcat populations. If habitat conditions (inc. prey availability) are suitable to sustain a healthy female wildcat population, a cohesive wildcat population would be expected, with potentially low permeability to domestic cats. Such scenario could buffer the effects of feral and hybrid cats, minimizing introgression potential. This assertion is coherent with the results from other studies in the Iberian region, where wildcat genetic integrity is maintained when habitat conditions are favorable, even under close contact with domestic cats (Gil‐Sanchez et al., 2015; Oliveira, Godinho, Randi, & Alves, 2008). Under Hubbs’ “desperation hypothesis” (Hubbs, 1955), restricted mate options resultant from depleted populations may promote mating with heterospecifics, leading to hybridization (Bohling & Waits, 2015; McCracken & Wilson, 2011). Therefore, under centuries‐long of continued contact between wild and domestic cats in Iberia, it is plausible to depict a scenario by which the relative skewness in the frequency of interbreeding events toward male wildcats with female domestic cats leads to the currently observed limited introgression of domestic genes into Iberian wildcat populations (Mattucci et al., 2016). It is possible that such hybridization directionality may be driven by the stricter habitat requirements of female wildcats. Although our results do not allow testing the causational link between habitat quality and the rate of hybridization between wild and domestic cats, they certainly raise this hypothesis and pave way for future research directions about these eco‐evolutionary processes.

The dynamics of contact between domestic and wild counterparts also affects disease transmission, with downstream consequences for the wildlife conservation (Smith, Sax, & Lafferty, 2006). Domestic cats are thought to be the main reservoirs of viral pathogens that have been reported in several wildcat populations in Europe (e.g., Artois & Remond, 1994; Duarte, Fernandes, Santos, & Tavares, 2012; Millán & Rodríguez, 2009). However, the spillover of diseases from domestic to wildcat populations, largely neglected, deserve urgently to be studied as they may also have important consequences for the conservation of the European wildcat.

## 5 CONCLUDING REMARKS

Using a multipopulation approach, we explicitly handle the sex‐specific behavioral choices of the European wildcat expressed through differential spatial ecology and hypothesize that such specificities could entail important eco‐evolutionary consequences.

Our results suggest that females have stricter habitats requirements and are less tolerant to humans at the landscape level than males, which have important implications for the conservation and management of European wildcat populations. This prudent behavior of females provides them with the best reproductive and foraging options by reducing energetic costs, increasing accessibility to prey, and securing adequate breeding grounds (Gittleman & Harvey, 1982; Gompper & Gittleman, 1991). Several authors propose the restoration/preservation of ecological corridors to increase gene flow within biogeographic regions as a main conservation priority for the European wildcat (Klar et al., 2008; Mattucci et al., 2016; Say, Devillard, Léger, Pontier, & Ruette, 2012; Steyer, Johnson, Kitchener, & Macdonald, 2016), to promote the genetic rescue of small and inbred populations (Whiteley, Fitzpatrick, Funk, & Tallmon, 2015). Our study adds to these recommendations and indicate that conservation actions should target mid‐elevation areas with some degree of topographic complexity, where patches of broadleaf forests and scrublands should be promoted, and be large enough to accommodate a viable number of females. This would ensure the long‐term persistence of healthy and resilient source populations of European wildcats. Contact between populations should then be secured through the establishment and maintenance of effective ecological corridors, namely through the Natura 2000 network, to allow the dispersal of individuals.

Additionally, our study highlights the ecological relevance of considering sex‐related differences in environmental preferences to accurately address conservation issues. To our knowledge, this is the first study to cope with sex‐specific habitat selection patterns at a multipopulation scale of the European wildcat in the human‐dominated landscapes of Western Europe.

## CONFLICT OF INTEREST

None declared.

## AUTHOR CONTRIBUTIONS

PM and PF originally formulated the idea; TO, FU, JML‐M, EB‐D, JMB‐A, MM, JMG‐S, FD‐R, PF, and PM conducted fieldwork and generated telemetry data; TO and PM performed statistical analyses; TO and PM wrote the manuscript; and all authors contributed critically to the drafts and gave final approval for publication.

## DATA ACCESSIBILITY

The data used for this study are archived in Dryad Digital Repository by Oliveira et al. (2018).
