# Supplementary material for: Females know better: Sex‐biased habitat selection by the European wildcat
Source: Ecol Evol. 2018 Aug 29;8(18):9464–77. doi: 10.1002/ece3.4442 (PMC6194279; doi:10.1002/ece3.4442)
Supplement: Supplementary file 1 [file ECE3-8-9464-s001.docx]

*Ecology and Evolution*

Supplementary Material

**Females know better: Sex-biased habitat selection by the European wildcat**

Teresa Oliveira, Fermín Urra, José María López-Martín, Elena Ballesteros-Duperón, José Miguel Barea-Azcón, Marcos Moléon, José María Gil-Sánchez, Paulo C. Alves, Francisco Díaz-Ruiz, Pablo Ferreras and Pedro Monterroso

**Appendix S1: Animal trapping, handling and radio-tracking procedures**

*Guadiana Valley Natural Park (GVNP)*

Animals were captured with 13 box-traps (180 x 80 x 80 cm), using Iberian lynx *Lynx pardinus* urine and live house pigeons (*Columba* sp.), unavailable to captured animals, as lure. Box-traps were checked daily after sunrise, in order to minimize animal stress. The trapping session took 377 trapping days, between July-August 2004, with a total of 6 animals captured (two males and four females). Captured animals were chemically immobilized with a combination of medetomidine hydrochloride (Domitor, Espoo, Finland; 0.1mgmL^-1^) and ketamine hydrochloride (Imalgene, Lyon, France; 1 gmL^-1^), with average dosages of 0.06 (SE 0.01) mgKg^-1^and 3.08 (SE 0.45) mgKg^-1^, respectively. Animals were weighted, sexed and checked for any injury and sanitary disorder. Blood samples were also collected for genetic confirmation of the taxonomic classification.

Individuals were fitted with Wildlife Materials Inc. (Murphysboro, IL, USA) HLPM 3320 radio-collars (80 g, approximate weight, frequencies ranging from 150 to 151MHz). Tagged animals were located by signal triangulation with the help of a four segment directional antenna (Telonics, Mesa, AZ, USA, model RA-14) and a portable receiver (Yaesus, Cypress, CA, USA, model FT-290RII), and bearings were determined using a handheld global positioning system unit equipped with an electronic compass (Garmin, Olathe, KS, USA,model E-Trex Summit). Triangulation was performed by a single researcher at different times of the day in order to cover the entire circadian cycle, performing occasionally tracking cycles. All animals were captured under official permit for the project “Distribuição, selecção de habitat e actividade do gato-bravo (*Felis silvestris*) no Parque Natural do Vale do Guadiana” [Distribution, habitat selection and activity of the European wildcat in the Parque Natural do Vale do Guadiana], emitted by the Instituto de Conservação da Natureza [Nature Conservation Institute]. For further details, see Monterroso et al. (2009).

*Izagaondoa Valley*

Animals were captured with 14 box-traps (130 x 60 x 45 cm), using live house pigeons (*Columba* sp.) and dwarf hens, unavailable to captured animals, as lure. All box-traps were checked daily, and were not active between April-June, due to the possibility of kitten’s raising. Two trapping sessions were performed, between February-March 1996 and March-July 1996, taking 164 and 2689 trapping days, respectively, with a total of 4 animals captured (two males and two females). Another 3 animals were fitted with radio collars (two males and one females), although not captured during trapping season.

Captured animals were chemically immobilized with a combination of Tiletamina and Zolazepam (1:1; Zoletil, Virvac S. A., Espluges de Llobregat, Barcelona, España), with average dosages of 14.02 mg Zoletil/Kg. Animals were weighted, sexed and checked for any sanitary disorder. Blood samples were also collected for genetic confirmation of the taxonomic classification.

Individuals were fitted with Wildlife Materials Inc. (Murphysboro, IL, USA) radio-collars with frequencies ranging from 150 to 152MHz. Tagged animals were located by signal triangulation with the help of Yagi antennas (several models and brands) and a portable receiver (AVM Inst. LA-12), and bearings were determined using a hand compass. Triangulation was at different times of the day. For further details, see Urra (2003).

*Lleida Region*

Animals were captured with 12 box-traps (180 x 80 x 80 cm), using and live house pigeons (*Columba* sp.), unavailable to captured animals, as lure. Box-traps were checked daily after sunrise, in order to minimize animal stress. The trapping session took 1,004 trapping days, between November 1999 and October 2000, with a total of 9 animals captured (six males and three females). Captured animals were chemically immobilized with a combination of medetomidine hydrochloride (Domitors, Espoo, Finland; 0.1mgmL^-1^) and ketamine hydrochloride (Imalgene, Lyon, France; 1 gmL^-1^), with average dosages of 0.28 (SE 0.21) mL and 0.19 (SE 0.20) mL, respectively. Animals were weighted, sexed and checked for any sanitary disorder. Blood samples were also collected for specific antibodies detection (Ac Feline Coronavirus, Ac Toxoplasma Igg, Ac Feline Immunodeficiency Virus, Ac Feline Leukemia Virus) and general hematological parameters.

Individuals were fitted with Biotrack Ltd. (Wareham, UK) TW3 radio-collars (100 g, approximate weight, frequencies ranging from 150 to 151MHz). Tagged animals were located by signal triangulation with the help of a yagi three segment direction antenna (Telonics, Mesa, AZ, USA) and a T-4 Telonics portable receiver (Telonics, Mesa, AZ, USA), and bearings were determined using a an electronic compass. A single researcher performed triangulation at different times of the day in order to cover the entire circadian cycle, performing occasionally tracking cycles.

*Cabañeros National Park (CNP)*

Animals were captured with 14 box-traps (four models: 105 x 38 x 38 cm, 145 x 36 x 55 cm, 152 x 45 x 50 cm and 95 x 45 x 50 cm), using live house pigeons (*Columba* sp.) and red-legged partridges (*Alectoris rufa*), unavailable to captured animals, as lure. Box-traps were checked daily after sunrise, in order to minimize animal stress. Three trapping sessions took a total of 791 trapping days, between March and December 2014. A total of 5 wildcats were captured (3 males and 2 females), but only one male and one female were radio-collared. Captured animals were chemically immobilized with a combination of medetomidine hydrochloride (Medetor, Virbac, Barcelona, Spain; 1mgmL^-1^) and ketamine hydrochloride (Imalgene, Merial Lab., Barcelona, Spain; 0.1 gmL^-1^), with average dosages of 0.11 (SE 0.01) mgKg^-1^ and 5.62 (SE 0.49) mgKg^-1^, respectively. Animals were weighted, sexed and checked for any injury or sanitary disorder. Blood samples were also collected for genetic confirmation of the taxonomic classification.

Individuals were fitted with GPS-VHF radio-collars (Telenax, Mexico), with 90g of approximate weight andfrequencies ranging 150-151 MHz. Tagged animals were located through the collar GPS unit (only data from the female could be retrieved with 6.5% success rate) but also by signal triangulation with the help of a Yagi antenna and a portable receiver (R-1000 Telemetry Receiver, Communications Specialists, Inc., Orange, California, USA), and bearings were determined using a handheld global positioning system unit equipped with an electronic compass (model GPSMAP 60CS, Garmin, Olathe,KS,USA). Triangulation was performed at different times of the day in order to cover the entire circadian cycle. All animals were captured under official permit nr. 2701.03, emitted by the Castilla La-Mancha National Parks Management Commission. For further details, see Ferreras et al. 2016.

*Sierra Arana*

Animals were captured with box-traps (100 x 50 x 70 cm), using live house pigeons (*Columba* sp.), unavailable to captured animals, as lure. Box-traps were checked daily after sunrise, in order to minimize animal stress. The trapping session was performed between March-June 2003, taking 2006 trapping days, with a total of 11 animals captured (three males and eight females), although only the data from 6 animals (two males and four females) was considered for this study.

Captured animals were chemically immobilized with a combination of ketamine hydrochloride and xylazine, with average dosages of 10 mg/kg. Animals were weighted, sexed and checked for any sanitary disorder. Blood, hair and tissue samples were also collected for genetic confirmation of the taxonomic classification.

Individuals were fitted with Biotrack radio-collars, with frequencies ranging from 150 to 151MHz and with a weight of 40g. Tagged animals were located by signal triangulation with the help of Yagi antennas and a portable receiver (Falcon Five receiver, Wildlife materials), and bearings were determined using a hand compass. Triangulation was performed at different times of the day in order to cover the entire circadian cycle, performing occasionally tracking cycles. All animals were captured under official permit nr. SGMN/GyB/JMIF, emitted by the Consejería de Medio Ambiente de y Ordenación del Territorio of the Junta de Andalucía.

*References*

Monterroso, P., Brito, J.C., Ferreras, P., Alves, P.C., 2009. Spatial ecology of the European wildcat in a Mediterranean ecosystem: dealing with small radio-tracking datasets in species conservation. Journal of Zoology 279, 27-35.

Urra, F.n., 2003. El gato montés en Navarra: Distribución, Ecología y Conservación, In Facultad de Ciencias, Departamento de Ecología. p. 207. Universidad Autónoma de Madrid, Madrid.

Ferreras, P., Díaz-Ruiz, F., Alves, P.C., Monterroso, P., 2016. Factores de la coexistencia de mesocarnívoros en parques nacionales de ambiente mediterráneo. In: *Proyectos de Investigación en Parques Nacionales 2011-2014*: 321-339*.* P. Amengual (Ed.). Organismo Autónomo Parques Nacionales, MAGRAMA, Madrid.

**Appendix S2: Ecological Overview of the Study Areas**

**Table B1.** Quantitative information about ecological covariates (annual temperature, total precipitation, slope, elevation, land cover percentage, and minimum distance to permanent water sources, roads and human settlements) in each study area. With the exception of land cover and precipitation, all values present the mean value, with the respective range of values. Climate variables were obtained only for the period when animals were monitored.

| Study Area | Annual Temperature (ºC) | Annual Precipitation (mm) | Slope (º) | Elevation (m) | Land Cover (%) | | | Distance to Permanent Water Bodies (km) | Distance to Roads (km) | Distance to Human Settlements (km) |
| --- | --- | --- | --- | --- | --- | --- | --- | --- | --- | --- |
|  |  |  |  |  | Agr | BFr | Scr |  |  |  |
| GVNP | 17.10 [10.80 - 23.40] | 327.80 [316.50 - 339.10] | 8.12 [0.00 - 40.73] | 140.54 [10.83 - 347.17] | 35.57 | 5.83 | 46.99 | 0.89 [0.00 - 3.47] | 0.65 [0.00 - 2.58] | 2.05 [0.00 -5.31] |
| IZV | 13.30 [7.80 - 18.47] | 730.63 [583.70 - 823.90] | 12.48 [0.00 - 55.77] | 616.23 [419.03 - 1262.01] | 41.45 | 10.11 | 20.25 | 1.72 [0.01 - 7.28] | 1.04 [0.00 - 5.17] | 0.99 [0.00 - 4.00] |
| SA | 16.00 [9.20 - 22.80] | 351.15 [307.60 - 394.70] | 12.46 [0.00 - 57.01] | 1170.06 [880.00 - 1904.98] | 16.41 | 6.15 | 34.45 | 8.81 [5.82 - 11.50] | 2.04 [3.53 - 6.58] | 3.28 [0.00 - 7.75] |
| CNP | 16.60 [10.60 - 22.50] | 342.4 | 7.04 [0.00 - 40.78] | 712.37 [598.64 - 1007.00] | 7.8 | 20.51 | 33.56 | 2.94 [ 0.00 - 8.37] | 5.16 [0.01 - 9.71] | 4.16 [0.03 - 9.70] |
| LD | 16.40 [12.40 - 20.50] | 394.90 [385.2 - 404.60] | 9.93 [0.00 - 54.39] | 462.72 [289.36 - 757.07] | 50.14 | 17.02 | 18.87 | 0.89 [0.00 - 3.26] | 1.24 [0.00- 5.12] | 0.79 [0.00 - 3.07] |

**Appendix S3: European wildcat data**

**Table C1.** Overview of the captured wildcats and the available telemetry data obtained for each study area. Only individuals that achieved a reliable home range estimate are represented.

| **Study Area** | **Gender** | | **ID** | | **Age** | | | **Sampling Period** | | **Nr of locations** | | | **Home range Area (km^2^)** |
| --- | --- | --- | --- | --- | --- | --- | --- | --- | --- | --- | --- | --- | --- |
|  |  |  | |  | |  | **Beginning** | | **End** | |  |  | |
| **Izagaondoa**  **(Navarre, SP)** | F |  | | NAF01 | | Adult | 27-09-1996 | | 23-07-1998 | | 786 | 3.14 | |
|  | F |  | | NAF02 | | Sub-adult | 30-01-1997 | | 12-03-1998 | | 403 | 5.92 | |
|  | M |  | | NAM01 | | Adult | 01-02-1997 | | 28-04-1998 | | 173 | 41.21 | |
|  | M |  | | NAM02 | | Adult | 30-01-1998 | | 16-11-1998 | | 346 | 23.44 | |
|  | M |  | | NAM03 | | Sub-adult | 18-06-1997 | | 17-10-1997 | | 118 | 10.72 | |
| **Lleida**  **(Catalonia, SP)** | F |  | | CTF01 | | Adult | 13-04-1999 | | 11-04-2000 | | 128 | 4.94 | |
|  | M |  | | CTM01 | | Adult | 11-07-1999 | | 25-05-2000 | | 92 | 13.75 | |
|  | M |  | | CTM02 | | Adult | 13-08-1999 | | 11-09-1999 | | 39 | 5.53 | |
|  | M |  | | CTM03 | | Adult | 05-08-2000 | | 30-06-2000 | | 38 | 1.22 | |
|  | M |  | | CTM05 | | Adult | 05-07-1999 | | 30-06-2000 | | 121 | 17.63 | |
|  | M |  | | CTM06 | | Adult | 07-06-1999 | | 04-10-2000 | | 143 | 13.60 | |
| **Sierra Arana**  **(Andalusia, SP)** | M |  | | GRM02 | | Adult | 26-03-2003 | | 26-01-2004 | | 36 | 30.78 | |
|  | F |  | | GRF01 | | Adult | 25-06-2003 | | 01-05-2004 | | 61 | 13.99 | |
| **GVNP** | F |  | | GVF01 | | Adult | 22-07-2004 | | 21-12-2004 | | 38 | 3.68 | |
| **(Southeastern PT)** | F |  | | GVF03 | | Adult | 13-07-2004 | | 15-03-2005 | | 68 | 3.81 | |
|  | M |  | | GVM01 | | Adult | 16-08-2004 | | 20-04-2005 | | 73 | 14.68 | |
| **CNP** | M |  | | CBM01 | | Adult | 27-03-2014 | | 10-12-2014 | | 40 | 43.01 | |
| **(Castille-La Mancha, SP)** | F |  | | CBF01 | | Adult | 28-03-2014 | | 06-06-2014 | | 88 | 59.78 | |

**Appendix S4: Generalized Linear Mixed Models**

**Table D1.** Univariate Generalized Linear Mixed Models (GLMMs) for habitat selection at the landscape level for male and female European wildcats. Only uncorrelated covariates are included. *k* - Number of model parameters, *AICc* - Aikake Information Criterion corrected for small sample sizes

| **Gender** | **Covariate** | **Model** | **k** | **ΔAICc** |
| --- | --- | --- | --- | --- |
| **Females** | Distance to roads  Distance to permanent water sources | dR + dR^2^  dW + dW^2^ | 4  4 | 0.00  5702.93 |
|  | Distance to broadleaf forests | dBFr + dBFr^2^ | 4 | 13302.57 |
|  | Area occupied by agricultural lands | Agr + Agr^2^ | 4 | 14483.49 |
|  | Slope  Elevation | Slp + Slp^2^  Elv + Elv^2^ | 4  4 | 17178.39  17882.33 |
|  | Distance to scrublands | dScr + dScr^2^ | 4 | 18700.23 |
| **Males** | Distance to human settlements | dH + dH^2^ | 4 | 0 |
|  | Distance to permanent water sources | dW + dW^2^ | 4 | 812.42 |
|  | Distance to broadleaf forests  Area occupied by agricultural lands | dBFr + dBFr^2^  Agr + Agr^2^ | 4  4 | 929.23  1302.17 |
|  | Distance to scrublands | dScr + dScr^2^ | 4 | 1425.91 |
|  | Slope | Slp | 3 | 1556.02 |

**Table D2.** Univariate Generalized Linear Mixed Models (GLMMs) for habitat selection at the home range level for male and female European wildcats. Only uncorrelated covariates are included. *k* - Number of model parameters, *AICc* - Aikake Information Criterion corrected for small sample sizes

| **Gender** | **Covariate** | **Model** | **k** | **ΔAICc** |
| --- | --- | --- | --- | --- |
| **Females** | Area occupied by scrublands Distance to human settlements | Scr + Scr^2^  dH + dH^2^ | 4  4 | 0.00  43.74 |
|  | Distance to permanent water sources | dW | 3 | 45.84 |
|  | Area occupied by broadleaf forests | BFr | 3 | 49. 31 |
|  | Slope | Slp + Slp2 | 4 | 65.22 |
| **Males** | Slope | Slp + Slp^2^ | 4 | 0.00 |
|  | Area occupied by agricultural lands | Agr | 3 | 8.61 |
|  | Distance to broadleaf forests | dDFr + dDFr^2^ | 4 | 10.37 |
|  | Distance to scrublands  Distance to permanent water sources | dScr + dScr^2^  dW + dW^2^ | 4  4 | 13.08  14.01 |
|  | Distance to roads | dR | 3 | 19.89 |

**Table D3.** Model averaged covariate coefficients of the Generalized Linear Mixed Effects Models (GLMMs) for habitat selection at the landscape and home range level by female and male European wildcats. β┴^- Parameter estimate, SE - Standard Error, CI95 - 95% Confidence interval for the parameter estimate. Bold values represent statistically significant coefficients. Agr- Area occupied by agricultural lands; dAgr- distance to agricultural lands; dDFr- distance to deciduous forests; DFr- area occupied by deciduous forests; Scr- areas area occupied by scrublands; dScr- distance to scrublands; Slp- Slope; Elv- elevation; dR – distance to roads; dW – distance to permanent water sources; dH – distance to human settlements

|  |  | **Landscape Level** | | | | | | |  | **Home Range Level** | | | | | | |
| --- | --- | --- | --- | --- | --- | --- | --- | --- | --- | --- | --- | --- | --- | --- | --- | --- |
|  |  | **Females** | | |  | **Males** | | |  | **Females** | | |  | **Males** | | |
| **Covariate** |  | $\overset{^}{\boldsymbol{\beta}}$ | **SE** | **CI_95_** |  | $\overset{^}{\boldsymbol{\beta}}$ | **SE** | **CI_95_** |  | $\overset{^}{\boldsymbol{\beta}}$ | **SE** | **CI_95_** |  | $\overset{^}{\boldsymbol{\beta}}$ | **SE** | **CI_95_** |
| **Intercept** |  | 3.86 | 3.35 | [-2.70 ; 10.43] |  | 0.57 | 0.40 | [-0.21; 1.35] |  | **-2.26** | **0.03** | **[-2.70; -1.81]** |  | **-2.33** | **0.04** | **[-2.44; -2.22]** |
| **Agr** |  | **0.38** | **0.06** | **[0.27 ; 0.50]** |  | **0.18** | **0.02** | **0.15; -0.22]** |  | - | - | - |  | **-0.07** | **0.04** | **[-0.15; -0.01]** |
| **Agr^2^** |  | **-0.33** | **0.06** | **[-0.43 ; -0.24]** |  | **-0.32** | **0.03** | **[-0.36; -0.28]** |  | - | - | - |  | - | - | - |
| **dAgr** |  | - | - | - |  | - | - | - |  | - | - | - |  | - | - | - |
| **dAgr^2^** |  | - | - | - |  | - | - | - |  | - | - | - |  | - | - | - |
| **dBFr** |  | **-2.96** | **0.08** | **[-3.12; -2.80]** |  | **-0.59** | **0.03** | **[-0.65; -0.53]** |  | - | - | - |  | -0.14 | 0.07 | [-0.28; 0.00] |
| **dBFr^2^** |  | **0.52** | **0.03** | **[0.47; 0.58]** |  | **0.08** | **0.01** | **[0.05; 0.10]** |  | - | - | - |  | 0.02 | 0.01 | [-0.01; 0.03] |
| **BFr** |  | - | - | - |  | - | - | - |  | **0.21** | **0.03** | **[0.15; 0.27]** |  | - | - | - |
| **Scr** |  | - | - | - |  | - | - | - |  | **0.27** | **0.03** | **[0.20; 0.33]** |  | - | - | - |
| **Scr^2^** |  | - | - | - |  | - | - | - |  | 0.01 | 0.03 | [-0.02; 0.03] |  |  |  |  |
| **dScr** |  | **-0.35** | **0.05** | **[-0.45; -0.25]** |  | **-0.05** | **0.02** | **[-0.09; -0.01]** |  | - | - | - |  | -0.01 | 0.06 | [-0.11; 0.11] |
| **dScr^2^** |  | **0.08** | **0.05** | **[0.04; 0.12]** |  | -0.03 | 0.01 | [-0.04; 0.01] |  | - | - | - |  | **-0.04** | **0.03** | **[-0.09; -0.01]** |
| **dH** |  | - | - | - |  | **1.00** | **0.03** | **[0.94; 1.06]** |  | **-0.38** | **0.09** | **[-0.55; -0.21]** |  | - | - | - |
| **dH^2^** |  | - | - | - |  | **0.20** | **0.02** | **[0.17; 0.23]** |  | 0.02 | 0.02 | [-0.02 ; 0.04] |  | - | - | - |
| **dR** |  | **5.35** | **0.09** | **[5.18; 5.52]** |  | - | - | - |  | - | - | - |  | - | - | - |
| **dR^2^** |  | **0.58** | **0.06** | **[0.46; 0.70]** |  | - | - | - |  | - | - | - |  | - | - | - |
| **dW** |  | **0.70** | **0.06** | **[0.59; 0.82]** |  | **-0.54** | **0.03** | **[-0.60; -0.49]** |  | - | - | - |  | **-0.19** | **0.06** | **[-0.30; -0.08]** |
| **dW^2^** |  | **-2.76** | **0.06** | **[-2.88; -2.64]** |  | **-0.48** | **0.03** | **[-0.53; -0.43]** |  | - | - | - |  | **0.06** | **0.02** | **[0.01; 0.10]** |
| **Slp** |  | **0.36** | **0.04** | **[0.28; 0.44]** |  | **-0.06** | **0.02** | **[-0.09; -0.03]** |  | -0.01 | 0.02 | [-0.03 ; 0.03] |  | **0.12** | **0.05** | **[0.03; 0.15]** |
| **Slp^2^** |  | -0.02 | 0.03 | [-0.07; 0.03] |  | - | - | - |  | - | - | - |  | -0.04 | 0.02 | [-0.08; 0.01] |
| **Elv** |  | **-0.72** | **0.07** | **[-0.87; -0.58]** |  | - | - | - |  | - | - | - |  | - | - | - |
| **Elv^2^** |  | **-0.67** | **0.05** | **[-0.77; -0.58]** |  | - | - | - |  | - | - | - |  | - | - | - |
